# Airway and parenchyma transcriptomics in a house dust mite model of experimental asthma

**DOI:** 10.1186/s12931-022-02298-x

**Published:** 2023-01-25

**Authors:** Xiaofan Tu, Henry M. Gomez, Richard Y. Kim, Alexandra C. Brown, Emma de Jong, Izabela Galvao, Alen Faiz, Anthony Bosco, Jay C. Horvat, Philip Hansbro, Chantal Donovan

**Affiliations:** 1 Priority Centre for Healthy Lungs, Hunter Medical Research Institute, The University of Newcastle, Newcastle, NSW Australia; 2 Faculty of Science, School of Life Sciences, University of Technology Sydney, Sydney, NSW Australia; 3 Centre for Health Research, Telethon Kids Institute, The University of Western Australia, Nedlands, WA Australia; 4 Centre for Inflammation, Faculty of Science, School of Life Sciences, Centenary Institute and University of Technology Sydney, Sydney, NSW Australia; 5 Asthma and Airway Disease Research Center, University of Arizona, Arizona, USA

**Keywords:** House dust mite, Asthma, Remodeling, AHR, Transcriptomics, SPI1

## Abstract

Lung transcriptomics studies in asthma have provided valuable information in the whole lung context, however, deciphering the individual contributions of the airway and parenchyma in disease pathogenesis may expedite the development of novel targeted treatment strategies. In this study, we performed transcriptomics on the airway and parenchyma using a house dust mite (HDM)-induced model of experimental asthma that replicates key features of the human disease. HDM exposure increased the expression of 3,255 genes, of which 212 were uniquely increased in the airways, 856 uniquely increased in the parenchyma, and 2187 commonly increased in both compartments. Further interrogation of these genes using a combination of network and transcription factor enrichment analyses identified several transcription factors that regulate airway and/or parenchymal gene expression, including transcription factor EC (TFEC), transcription factor PU.1 (SPI1), H2.0-like homeobox (HLX), metal response element binding transcription factor-1 (MTF1) and E74-like factor 4 (ets domain transcription factor, ELF4) involved in controlling innate immune responses. We next assessed the effects of inhibiting lung SPI1 responses using commercially available DB1976 and DB2313 on key disease outcomes. We found that both compounds had no protective effects on airway inflammation, however DB2313 (8 mg/kg) decreased mucus secreting cell number, and both DB2313 (1 mg/kg) and DB1976 (2.5 mg/kg and 1 mg/kg) reduced small airway collagen deposition. Significantly, both compounds decreased airway hyperresponsiveness. This study demonstrates that SPI1 is important in HDM-induced experimental asthma and that its pharmacological inhibition reduces HDM-induced airway collagen deposition and hyperresponsiveness.

**Supplementary Information:**

The online version contains supplementary material available at 10.1186/s12931-022-02298-x.

## Introduction

Asthma is a chronic inflammatory lung disease with common symptoms of wheezing and shortness of breath associated with airway remodeling and hyperresponsiveness (AHR) [1–3]. Current therapies including bronchodilators and corticosteroids can be effective at managing symptoms and newer type 2-targeted biologics, such as duplizumab, may also be effective in subsets of patients with type 2-driven disease [4]. However, gaps remain in our understanding of what drives key disease features and current therapies may not be effective in all patients.

Transcriptomic studies in asthma have provided valuable information in the whole lung context [5–7], however, deciphering the individual contributions of the airway and parenchyma in disease pathogenesis may expedite the development of novel tissue-specific treatment strategies. A significant challenge in the study of gene expression patterns is deciphering the relationships between a large number of differentially expressed genes and overall functional effects. To address this, a network-based approach is being increasingly adopted to systematically explore the complex and dynamic transcriptional changes underlying diseases such as asthma and chronic obstructive pulmonary disease [8–13]. By utilizing bioinformatics tools such as weighted gene correlation network analysis (WGCNA) [14], co-expression network analysis can provide a holistic view of the global connectivity structure and functional organization of the gene expression program and identify subtle disease-associated changes in correlation structure that are not detected by conventional differential gene expression analysis [11, 15]. Furthermore, since disease-associated genes often interact to form disease modules [8], insights gained from co-expression network analysis can be leveraged to infer upstream module-driving genes that control entire disease pathways and can become promising therapeutic targets [11, 16].

In this study, we performed airway and parenchyma transcriptomics using a house dust mite (HDM)-induced model of experimental asthma that replicates key features of the human disease [17, 18]. Interrogation of HDM-induced genes using WGCNA upstream module analysis identified several transcription factors that regulate airway and/or parenchymal gene expression, including transcription factor PU.1 (SPI1). SPI1 inhibition using specific small molecule inhibitors, DB1976 or DB2313, had no protective effects on airway inflammation. However, DB2313 (8 mg/kg) decreased airway mucus secreting cell numbers, and DB2313 (1 mg/kg) and DB1976 (2.5 mg/kg and 1 mg/kg) reduced small airway collagen deposition. Both compounds decreased AHR. This study reveals important differences between the airway and parenchyma transcriptomic profiles in a HDM-induced model of asthma. We also demonstrate that SPI1 is important in HDM-induced experimental asthma pathogenesis and that its pharmacological inhibition reduces HDM-induced collagen deposition and AHR.

## Materials and methods

### Mice and ethics

Female wild-type BALB/c mice (Animal Resource Centre, Western Australia) were housed under specific pathogen-free conditions in the Hunter Medical Research Institute, Australia PC2 facility and maintained on a 12-hour day-night cycle with water and regular chow available *ad libitum*. Female BALB/c mice were chosen for this study due to their increased susceptibility to allergic airway inflammation induced by HDM [19]. All studies were performed under protocols approved by the Animal Care and Ethics Committee at the University of Newcastle.

### Model and treatments

Mice were treated intranasally with house dust mite (HDM; *Dermatophagoides pteronyssinus*) extract (25 µg in 30 µL phosphate buffered saline [PBS]; 15G10, Citeq Biologics) or saline (vehicle), 5 days per week for 3 weeks [17, 18]. Some groups of HDM-treated mice were treated with DB1976 (1 mg/kg or 2.5 mg/kg in PBS; #HY-135,797 A, MedChemExpress) or DB2313 (1 mg/kg or 8 mg/kg in PBS; #AOB33333, Aobious).

### Bronchoalveolar lavage, mucus-secreting cells (MSCs) and collagen deposition

Bronchoalveolar lavage fluid was collected as previously described [20–23]. MSCs were stained and counted as previously described [17, 24, 25]. Collagen was stained and analyzed as previously described [17, 18, 23, 25]. Additional details are provided in the online supplement.

### qPCR

Total RNA was isolated from homogenized lung tissue with TRIzol® Reagent (Invitrogen™) [26–28]. Random-primed reverse transcriptions were performed followed by real-time qPCRs. Spi1 (Forward primer: 5’-AAGCAGGGGATCTGACCAAC-3’; Reverse primer: 5’-AAGTCATCCGATGGAGGGGC-3’) gene expression was normalized to the expression of the transcript of the housekeeping gene hypoxanthine guanine phosphoribosyltransferase (*Hprt*) (Forward primer: 5’-AGGCCAGACTTTGTTGGATTTGAA-3’; Reverse primer: 5’-CAACTTGCGCTCATCTTAGGCTTT-3’). All reactions were performed using BioScript™ reverse transcriptase in 1x first-strand buffer according to the manufacturer’s instructions (Bioline). Real-time qPCR assays were performed with SYBR Green Supermix (KAPA Biosystems) and a Mastercycler® ep realplex2 system (Eppendorf South Pacific).

### Lung function

Airway hyperresponsiveness was assessed using the forced oscillation technique on a Scireq Flexivent FX1 machine as previously described [29, 30]. Additional details are provided in the online supplement.

### RNA extraction, bioanalysis, RNA-sequencing, differential gene expression and network analysis

Lung airway and parenchyma tissues were separated by blunt dissection under a dissection microscope as previously described [22]. Details for RNA extraction, bioanalysis, RNA-sequencing and analysis are provided in the online supplement. Differential gene expression and network analysis were performed as previously described [16, 31, 32] and details are provided in the online supplement.

### Statistics

Comparisons between two groups were performed using an unpaired t-test. Comparisons between multiple groups were carried out using one-way ANOVA with Sidak’s *post-hoc* test. AHR data was analyzed using two-way ANOVA with Bonferroni *post-hoc* test. All statistical analyses were carried out using Prism software (GraphPad version 9.0.0) and data are presented as mean ± SEM. A *P* value < 0.05 was considered statistically significant.

## Results

### Airway and parenchyma RNA-seq from HDM- and PBS-exposed mice reveals distinct gene expression in different tissue compartments

**Fig. 1:**
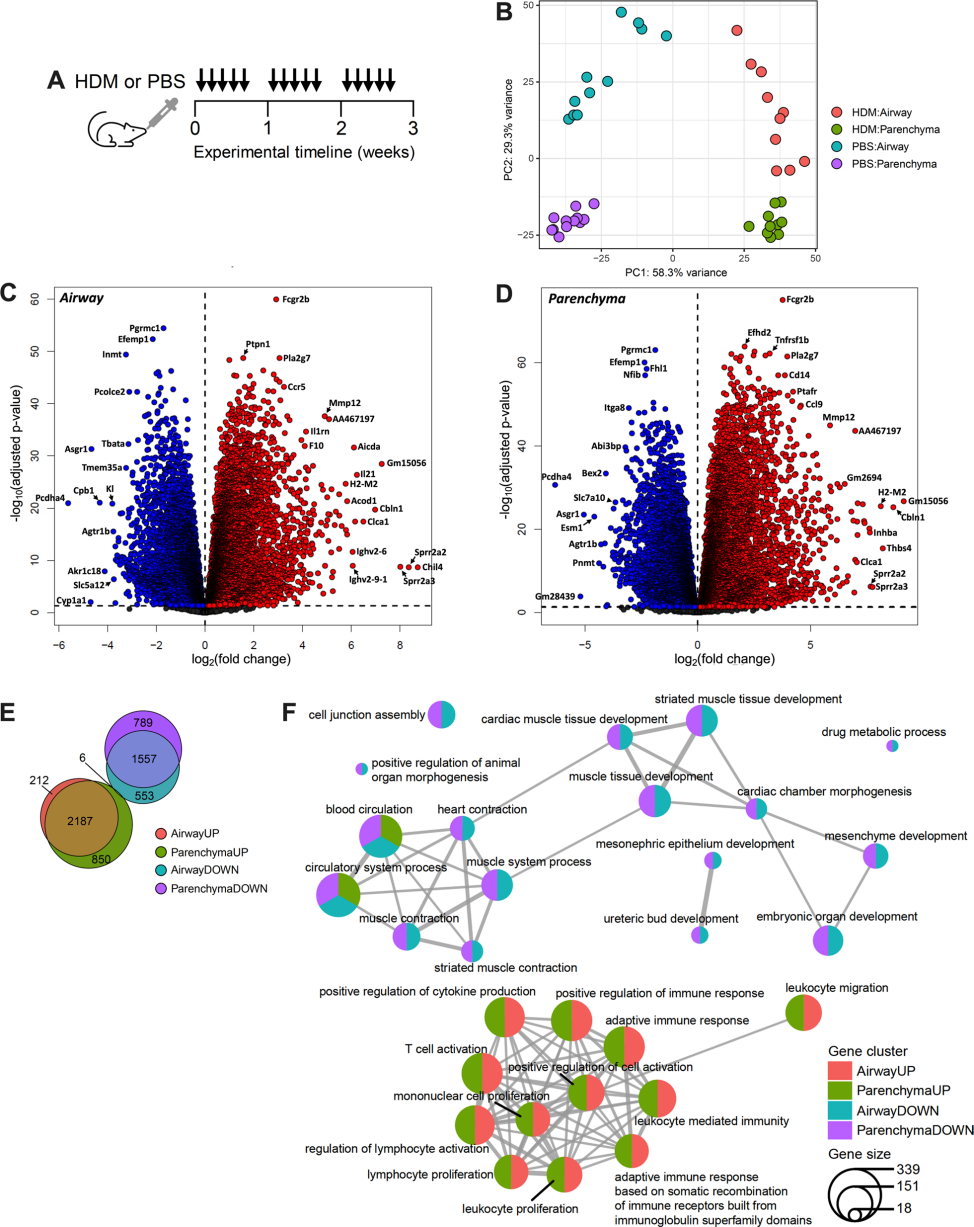
Airway and parenchyma RNA-seq from HDM- and PBS-exposed mice reveals compartment- and exposure-specific gene expression. **A** HDM (25 µg/30 µL) or PBS (30 µL) was administered intranasally 5 days/week for 3 weeks. **B** Principal component analysis plot of HDM and PBS airway and parenchyma samples HDM (*n* = 10) and PBS (*n* = 12). Volcano plots of upregulated (red) and downregulated (blue) gene transcripts in airway (**C**) and parenchyma (**D**), dotted line represents *P* = 0.05. **E** Venn diagram of up- and down-regulated genes in the airways and parenchyma. **F** Gene enrichment analysis in the airways and parenchyma

Airway and parenchyma tissues from mice exposed to HDM were subjected to bulk RNA-Seq and compared to PBS vehicle exposed controls (Fig. 1A). Principal component analysis revealed distinct transcriptomic signatures associated with each type of exposure and also distinct separations between the airway and parenchyma within each exposure type (Fig. 1B). mRNAs with altered expression in airway (Fig. 1C) and parenchyma (Fig. 1D) in HDM-exposed mice compared to PBS controls were visualized by volcano plots. Combining the lists of mRNAs with altered expression in airway and parenchyma compartments revealed that HDM exposure resulted in 3,255 upregulated genes and 2905 downregulated genes (log2FC ≥ 1 or ≤ -1, adj.p.val < 0.05) (Fig. 1E). Of these genes, 212 and 856 were upregulated, and 559 and 789 downregulated, in airways and parenchyma, respectively (Fig. 1E). There were 6 common genes that were upregulated in parenchyma and downregulated in airways. Gene enrichment analysis showed similar GO biological pathways upregulated in airways and parenchyma, including leukocyte migration and regulation of cytokine responses, whereas similar downregulated pathways included muscle development and muscle system processes. Furthermore, gene cluster changes in blood circulation and circulatory system processes were found to be up- and down-regulated in parenchyma samples and downregulated in airway samples (Fig. 1F). These data demonstrate key differences between mRNA expression in airway and parenchyma following HDM exposure.

### Network analysis identifies common and distinct upstream module-driving genes in airways and parenchyma from HDM- compared to PBS-exposed mice

**Fig. 2:**
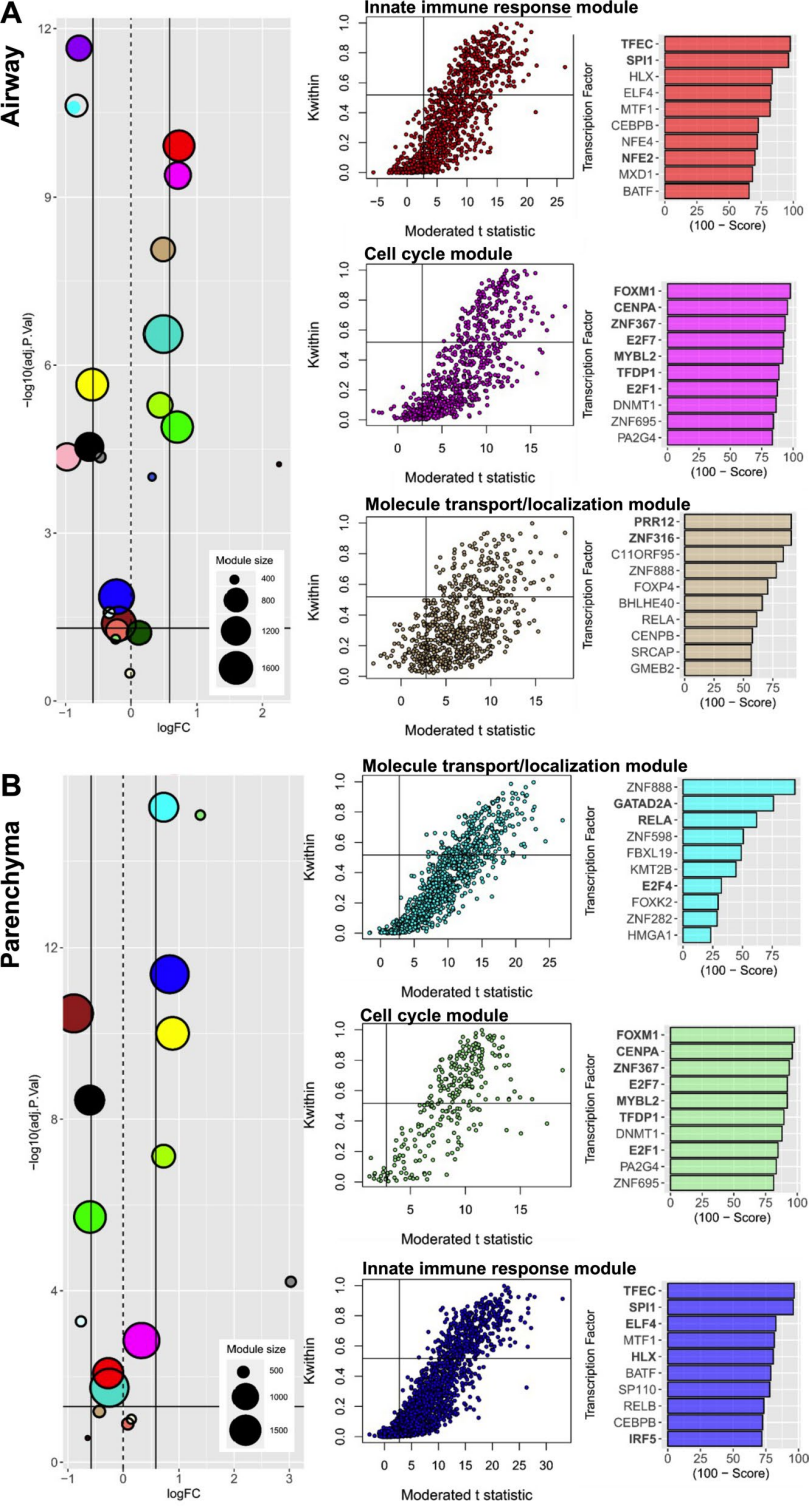
Upstream module-driving genes in the airways and parenchyma from HDM- compared to PBS-exposed mice. **A** Airway and **B** parenchyma compartments from HDM- (*n* = 10) compared to PBS-exposed mice (*n* = 12). Bubble plots for each differential gene expression cluster where the median adjusted p-value for all genes within a module was plotted against the median log fold-change, bubble size is representative of the number of genes within a module. ChIP-X Enrichment Analysis 3 (ChEA3) of the top ten predicted transcription factors with significant overlap with our driver gene signatures for **A** airway and **B** parenchyma compartments from HDM- (*n* = 10) compared to PBS-exposed mice (*n* = 12)

A powerful way to identify disease-associated biological mechanisms is to define the intersection between disease-associated gene networks and differential gene expression. By overlaying functionally distinct gene modules identified by network analysis with differentially expressed genes, disease-associated modules in different settings can be defined. To visualize this, bubble plots for each differential gene expression cluster were generated where the median adjusted p-value for all genes within a module was compared against the median log fold-change, and the bubble size representative of the number of genes within a module for the airways (Fig. 2A, Additional file 1: Table S1) and parenchyma (Fig. 2B, Additional file 1: Table S1). Having identified specific disease-associated network modules, we next examined the transcription factors responsible for driving these transcriptional changes. We identified a driver gene signature for each disease-associated module by selecting the genes with a module connectivity greater than 2 times the standard deviation from the mean, and with an adjusted p-value < 0.01 in terms of differential gene expression. We then used ChIP-X Enrichment Analysis 3 (ChEA3), which leverages prior knowledge about transcription factor targets assembled multiple resources including ChIP-seq experiments, to identify the top 10 predicted transcription factors for which their downstream targets had a significant overlap with our driver gene signatures. The three largest modules in the airways and parenchyma were innate immune, cell cycle and molecule transport/localization responses. Interestingly, the top 10 transcription factors by mean rank were similar in the cell cycle modules in the airway and parenchyma, however, there were differences in their rank order (Fig. 2A, B). Together, these data demonstrate key differences in predicted transcription factors that drive HDM-induced disease responses in the airways and parenchyma.

### SPI1 inhibition with DB1976 and DB2313 treatments has differential effects on weight loss and airway inflammation but inhibits airway remodeling

**Fig. 3:**
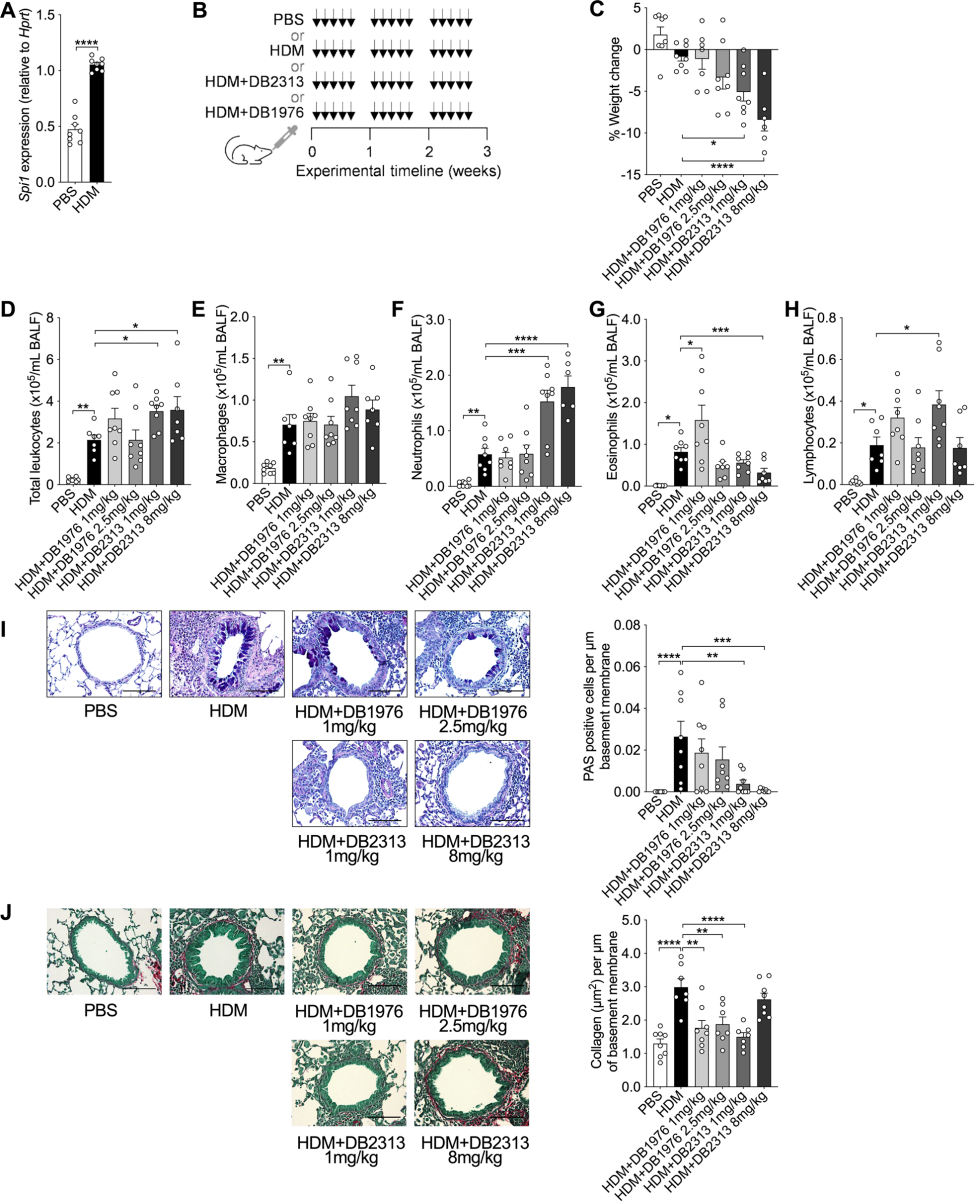
Pharmacological targeting of SPI1 affects weight loss, airway inflammation, and airway remodeling. **A** *Spi1* mRNA expression in PBS- and HDM-exposed mice. **B** Representative models of PBS (30 µL), HDM (25 µg/30 µL), HDM + DB1976 (1 mg/kg or 2.5 mg/kg) or HDM + DB2313 (1 mg/kg or 8 mg/kg) exposure intranasally 5 days/week for 3 weeks. **C** Percentage body weight of mice at the end of the model. **D** Total leukocytes, **E** macrophages, **F** neutrophils, **G** eosinophils and **H** lymphocytes in BALF. **I** AB-PAS staining for airway-associated mucus-secreting cells that were enumerated per µm of basement membrane. **J** Small airway collagen deposition measured using Sirius Red/Fast Green staining of lung sections and quantified per µm of basement membrane. All scale bars = 100 µm. Data expressed as mean ± SEM (n = 6–8 for each group). *P < 0.05, **P < 0.01, ***P < 0.001, ****P < 0.0001, A: unpaired t-test, C-J one-way ANOVA, Sidak’s *post hoc* test

SPI1 was one of the upstream transcription factors identified in our RNA-seq data that was predicted to be responsible for driving transcriptional changes in both the airways and parenchyma. Importantly, SPI1 also has commercially available pharmacological inhibitors. Therefore, we confirmed the increased mRNA expression of *Spi1* following 3-weeks of HDM exposure (Fig. 3A). We also interrogated *SPI1* expression in single-cell data from the integrated Human Lung Cell Atlas [33] and The Human Protein Atlas [34] and showed that *SPI1* expression is highest in lung macrophages (Additional file 1: Figure S1 and Additional file 1: Figure S2). Then, to assess the effects of therapeutically targeting SPI1 on HDM-induced experimental asthma, we administered DB1976 and DB2313, that interact with the DNA minor groove that flanks the PU.1 binding motif to inhibit its activity, throughout the model (Fig. 3B). HDM alone and HDM + DB1976 had minimal effects on body weight, however, HDM + DB2313 resulted in significant weight loss at both 1 mg/kg and 8 mg/kg compared to HDM alone (Fig. 3C).

HDM exposure increased inflammation in the airways and total leukocyte numbers in the BALF, consisting of macrophages, neutrophils, eosinophils and lymphocytes (Fig. 3D–H). DB1976 treatment largely had no effect on HDM-induced cellular inflammation, although treatment with 1 mg/kg increased eosinophil numbers (Fig. 3G). DB2313 treatment further increased HDM-induced total leukocytes (Fig. 3D), which were primarily neutrophils (Fig. 3F), and at 8 mg/kg reduced eosinophil numbers (Fig. 3G).

Exposure to HDM increased airway-associated mucus secreting cell numbers, which were reduced back to baseline levels with DB2313, but not DB1976, treatment at both doses (Fig. 3I). HDM exposure also increased airway fibrosis and small airway collagen deposition, and treatment with DB1976 (1 mg/kg and 2.5 mg/kg) and DB2313 (1 mg/kg) almost completely inhibited collagen deposition, which were at levels similar to baseline in PBS-exposed controls (Fig. 3J).

Together, these data demonstrate differential effects of DB1976 and DB2313 on HDM-induced airway inflammation and remodeling.

### DB1976 and DB2313 treatments inhibit HDM-induced AHR

**Fig. 4:**
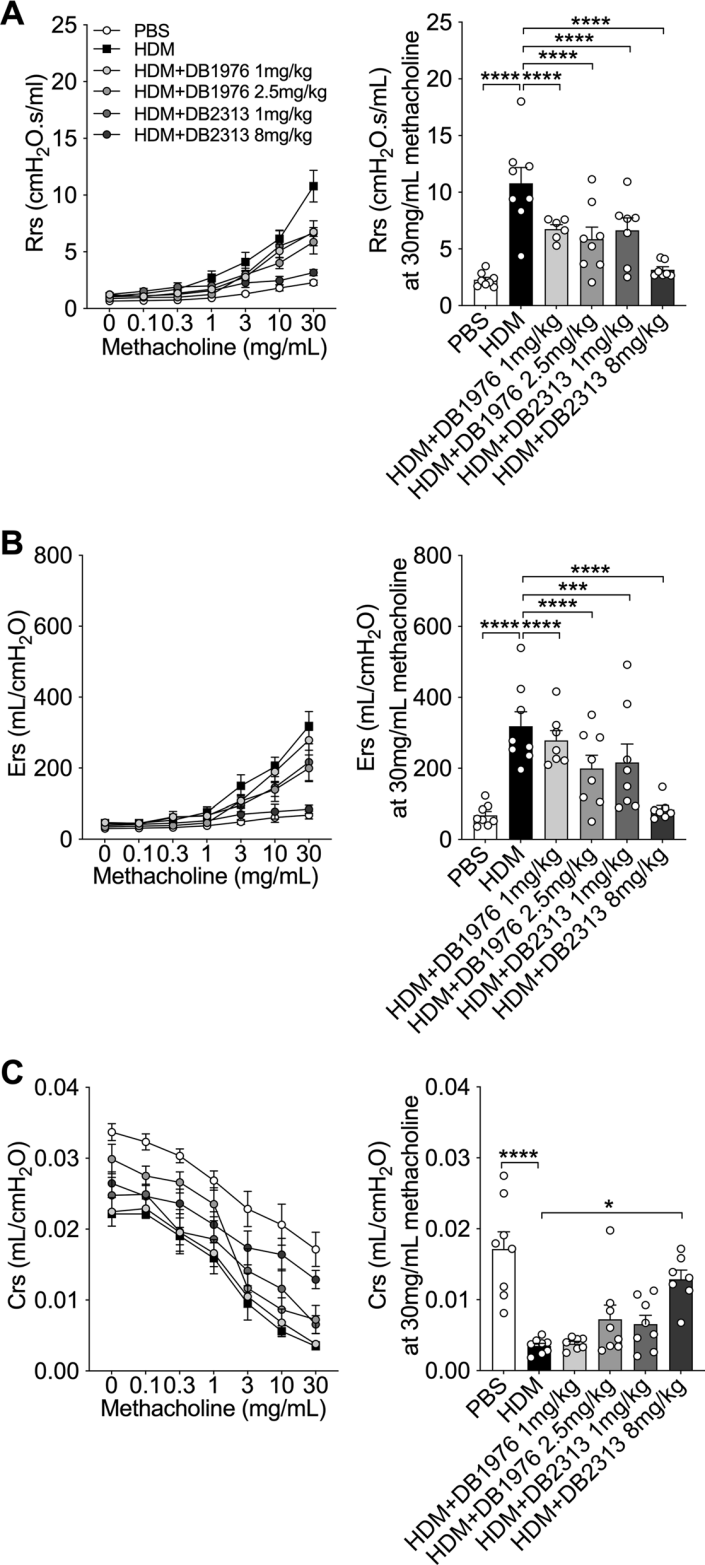
DB1976 and DB2313 treatments inhibit airway hyperresponsiveness in HDM-induced experimental asthma. Airway hyperresponsiveness measured in response to increasing doses of methacholine: **A** transpulmonary resistance (Rrs), **B** elastance (Ers) and **C** compliance (Crs). Data expressed as mean ± SEM (n = 6–8 for each group). *P < 0.05, ***P < 0.001, ****P < 0.0001. Two-way ANOVA, Bonferroni *post hoc* test

To assess the functional effects of targeting SPI1 on HDM-induced experimental asthma, we measured AHR following treatment with DB1976 and DB2313 throughout the model. HDM exposure substantially increased transpulmonary resistance (Rrs, Fig. 4A) and elastance (Ers, Fig. 4B), and decreased compliance (Crs, Fig. 4C) compared to PBS controls. Treatment with DB1976 (1 mg/kg and 2.5 mg/kg) and DB2313 (1 mg/kg and 8 mg/kg) decreased resistance and elastance. Treatment with DB2313 at 8 mg/kg completely inhibited the development of resistance and elastance and increased Crs back to baseline levels that were similar to those in PBS-exposed controls (Fig. 4C). Together these data show that DB1976 and especially DB2313 treatment inhibits HDM-induced AHR.

### Interrogation of downstream genes of SPI1 in human single cell data

We next used our mouse transcriptomics data to prioritise genes downstream of SPI1 with the highest logFC value in both airway and parenchyma between HDM and PBS mice and identified Interleukin 1 Receptor Antagonist (*Il1rn*), Platelet Activating Factor Receptor (*Ptafr*), Integrin Subunit Alpha X (*Itgax*), and C-type lectin domain family 7 member A (*Clec7a*). Significantly, cross-comparison with the Human Protein Atlas [34] shows that these genes are also highly co-expressed in human lung macrophages (Additional file 1: Figure S2).

## Discussion

The individual contributions of the airways and parenchyma in asthma pathogenesis is incompletely understood. Here, we performed transcriptomic analysis of the airways and parenchyma in a HDM-induced model of experimental asthma. Our approach, which was sufficiently powered (n = 10 per group) to assess upstream drivers of disease, highlights key similarities and differences between the airways and parenchyma. Notably, SPI1 was similarly upregulated in both the airways and parenchyma. Using pharmacological inhibitors of SPI1, DB1976 and DB2313, we show that treatment with these compounds has differential effects at different doses on airway-associated mucus secreting cell numbers and small airway collagen deposition, with no protective effects on airway inflammation. Significantly, both compounds at either dose decreased airway hyperresponsiveness, with DB2313 at 8 mg/kg having the strongest effect. Our study demonstrates the importance of assessing airway and parenchyma transcriptomics separately in HDM-induced experimental asthma. We define SPI1 as an important disease-associated transcription factor in both tissues and that its pharmacological inhibition may be a novel treatment strategy for the most intractable features of chronic asthma.

We first showed that the separation of airway and parenchyma data and in response to PBS or HDM on the PCA plot, are likely consequences of differences in the baseline gene expression profiles of the two tissue types, and HDM exposure. This is likely due to the different cellular compositions of these two compartments, including airway smooth muscle cells in the airway compartment, and ATII cells in the parenchyma. We next showed that there are distinctive sets of differentially expressed genes in the airways and parenchyma in HDM-induced experimental asthma. Gene cluster analysis showed that some similar pathways are upregulated in the airways and parenchyma, including leukocyte migration and regulation of cytokine responses, and these are comprised of numerous genes known to be involved in the early phase of allergen-induced airway inflammation [35]. Interestingly, downregulated pathways, including muscle development and muscle system processes, were found in both the airways and parenchyma. It is well established that airway smooth muscle plays key roles in asthma, including in airway hyperresponsiveness [36]. However, less is known about the role of vascular smooth muscle, although it has been shown that glucocorticoid treatment in asthma can increase airway vascular smooth muscle contraction and decrease airway blood flow [37]. Our analysis shows that the downregulation of genes involved airway and vascular smooth muscle development and processes may be common to both processes and dysregulated in asthma. Furthermore, gene cluster changes in blood circulation and circulatory system processes were found to be up- and down-regulated in parenchyma samples and downregulated in airway samples. These data highlight a complex relationship between vascular smooth muscle, blood circulation and circulatory system processes in experimental asthma that warrants further investigation.

We next utilized WGCNA to unveil mechanisms that may not be detected through standard differential gene expression analysis. The three largest median increase in expression with HDM exposure modules in the airways and parenchyma consisted of innate immune, cell cycle and molecule transport/localization responses. We then used ChEA3 [16] to rank transcription factors based on pooled information across six transcription factor target gene set libraries to identify those responsible for the observed changes in gene expression. Interestingly, the top 10 transcription factors by mean rank were similar in cell cycle modules in both the airways and parenchyma, however, there were some differences in the innate immune and molecule transport/localization responses in the airways and parenchyma.

We next interrogated the role of the transcription factor *Spi1* that is involved in the innate immune system module. Spi1 was upregulated in both the airways and parenchyma, and belongs to the erythroblast transformation specific (ETS) family of transcription factors that mediate a wide range of cellular processes, including growth, differentiation, signaling, and apoptosis [38]. *Spi1* also has known roles as a master regulator of hematopoiesis [39, 40], and is expressed in the respiratory tract, brain, endocrine tissues, gastrointestinal tract, liver and gallbladder, pancreas, kidney and urinary system, reproductive tract, muscle tissues, skin, bone marrow and lymphoid tissues. DB1976 and DB2313 are small molecule SPI1 inhibitors that are heterocyclic diamidines that bind with high specificity to AT-rich sequences in the DNA minor groove that is selective for the SPI1 protein and thus competitively inhibit SPI1 binding [41, 42]. In acute myeloid leukemia, inhibition of SPI1 using these inhibitors resulted in the downregulation of SPI1 downstream targets and reduced tumor burden [42]. Furthermore, another study showed that DB1976 treatment downregulated pro-fibrotic gene expression in fibroblasts and had both preventative as well as therapeutic benefits in a bleomycin-induced skin fibrosis model [43].

In our study, treatment with DB1976 increased airway eosinophils and decreased small airway collagen deposition but had no effect on mucus secreting cell numbers. In contrast, DB2313 increased neutrophil numbers, and reduced mucus secreting cell numbers and completely inhibited collagen (at 1 mg/kg but not 8 mg/kg). Significantly, treatment with both drugs at either dose reduced airway hyperresponsiveness, with almost complete inhibition with DB2313 at 8 mg/kg. SPI1 is proposed to modulate the expression of over 3,000 genes in hematopoietic cells [44–46], therefore the differences in the effects of DB1976 and DB2313 may be linked to the specific downstream genes that were regulated, such as *IL1RN*, *PTAFR*, *ITGAX* and *CLEC7A*. Interestingly, some studies show that *Spi1* can suppress Th2 cytokine expression, and that *Spi1* expression can be upregulated through selective antagonism of microRNA (miR)-126 [47–49]. Likewise, the deletion of miR-155 is associated with the upregulated expression of *Spi1* and reduced allergen-induced type 2 inflammation [50]. Whether these pathways are affected in HDM-induced experimental asthma warrant further investigation. Importantly, the expression of the majority of genes that can be regulated by *Spi1* can also be controlled by other transcription factors, which complicates measuring genes that are proposed to be *Spi1*-dependent as a readout of DB2313 and DB1976 target specificity. It is also important to consider that we administered DB2313 and DB1976 *via* the intranasal route to direct these compounds into the lungs, but we cannot exclude the possibility that these compounds entered the circulation and exerted effects on *Spi1* at peripheral sites. This is unlikely to affect the molecular specificity of these compounds to their target, but rather highlights that these extrapulmonary effects may promote feedback responses that exert effects on lung inflammation, structure, and function.

Whilst the effects of DB1976 and DB2313 on inflammation in HDM-induced experimental asthma were surprising, our data support the concept that inflammation, remodeling and AHR in allergic asthma can occur and be regulated independently [30, 51]. It is now well established that there is a disconnect between inflammation and AHR in asthma patients and that they are regulated by different processes. In support of our findings, a study of patients with chronic asthma showed no correlation between the numbers of inflammatory cells present in BALF and the level of AHR [52]. Our study, as well as those from others, provide strong evidence that airway remodeling can lead to constricted airways, which may drive the variation in AHR in asthma patients [52–54]. Currently, biologics for the treatment of severe asthma primarily focus on targeting inflammation. Our findings suggest that a broader set of drug targets remain to be discovered that can selectively target inflammation, remodeling, or both.

Interestingly, treatment with both DB1976 and DB2313 reduced features of airway remodeling and AHR, suggesting that HDM-induced Spi1 responses could drive these potentially inter-connected processes. Indeed, another study showed that DB1976 treatment of TGFβ-stimulated fibroblasts decreased *COL1A1*, α-SMA expression and F-actin [43], supporting the concept that Spi1 responses can promote remodeling. Taken together, the data show that it is likely that increased Spi1 responses can have context-dependent, dichotomous roles. Of note, our bioinformatics approach utilized a database of published findings to identify SPI1 as a key upstream driver of genes involved in the innate immune module, and our functional studies demonstrate that SPI1 is important in airway remodeling and AHR. Collectively, our data show that our approach has the capacity to identify new pathways and the functional effects of factors that may cross over several modules and can inform further studies that interrogate novel relationships. Future studies that examine the effects of different treatment regimens with DB1976 and DB2313, including treatment after establishment of disease, may provide additional insights into how these compounds affect remodeling and/or AHR.

A limitation of our study is the different high doses of DB1976 (2.5 mg/kg) and DB2313 (8 mg/kg) that were dictated by drug solubility and preclude direct dose-to-dose comparisons between the two highest doses of these drugs. However, at 1 mg/kg, the disparities in the physiological responses to DB1976 and DB2313 may partially be due to differences in their chemical structure and subsequent binding affinity. Indeed, reported IC_50_ values are lower for DB2313 compared to DB1976, indicating that DB2313 exhibits higher binding affinity for its target [42]. Whilst the selectivity of DB1976 and DB2313 for SPI1 has been established [41, 42], a rigorous examination of the specificity of these compounds against a large range of targets has not been performed. Differences in the specificity of these compounds could explain the differences in the physiological responses that were observed in vivo, and this will be important to examine in future studies. Additionally, DB1976 exhibits increased cellular toxicity in Lin^−^Sca1^+^c-Kit^+^ bone marrow cells from wild type mice compared to DB2313 [42], and DB2313 has more pronounced effects on reducing cell viability in THP-1 cells at high doses compared to DB1976 [42]. This suggests that high doses of these compounds have differential effects on cell viability depending on the type of cell that is exposed. However, which specific cell types are affected by intranasal drug administration in our model is unknown and a rigorous investigation of this is beyond the scope of our study but would be interesting to assess in future work by performing systemic, rather than lung local, administration. Furthermore, our study has used female mice, and it is well known that are sex-specific effects to allergen exposure [55], thus future studies examining the efficacy of DB1976 and DB2313 in male mice are warranted.

Our study demonstrates the advantages and value of using airway and parenchymal transcriptomics to assess and interrogate the roles of gene expression modules and upstream transcription factors at the lung local level. Importantly, we show that targeting one of these factors, Spi1, by direct administration of novel pharmacological agents allows us to delineate whether these modules are drivers or consequences of disease, and our data highlight the potential for targeting increased Spi1 responses in the lungs as a novel therapeutic strategy to reduce remodeling and AHR in asthma patients.

## Supplementary Information

**Additional file 1.** Online supplementary methods. **Table S1**: Airway and parenchyma network modules. **Figure S1**: SPI1 overlayed onto the single-cell data from the integrated Human Lung Cell Atlas. **Figure S2**: SPI1, IL1RN, PTAFR, ITGAX and CLEC7A overlayed onto the single-cell data from the integrated Human Protein Atlas.

## Data Availability

All data is available upon request. RNA-sequencing data are available through the NCBI GEO database (GSE199853).
