# Supplementary material for: Airway and parenchyma transcriptomics in a house dust mite model of experimental asthma
Source: Respir Res. 2023 Jan 25;24:32. doi: 10.1186/s12931-022-02298-x (PMC9878882; doi:10.1186/s12931-022-02298-x)
Supplement: Supplementary file 1 — Additional file 1. Online supplementary methods. Table S1: Airway and parenchyma network modules. Figure S1: SPI1 overlayed onto the single-cell data from the integrated Human Lung Cell Atlas. Figure S2: SPI1, IL1RN, PTAFR, ITGAX and CLEC7A overlayed onto the single-cell data from the integrated Human Protein Atlas. [file 12931_2022_2298_MOESM1_ESM.docx]

**Airway and parenchyma transcriptomics in a house dust mite model of experimental asthma**

Xiaofan Tu^1*^, Henry M. Gomez^1*^, Richard Y. Kim^1,2^, Alexandra C. Brown^1^, Emma de Jong^3^, Izabela Galvao^4^, Alen Faiz^2^, Anthony Bosco^5^, Jay C. Horvat^1^, Philip M. Hansbro^1,4*^, Chantal Donovan^1,2*^

^1^Priority Centre for Healthy Lungs, Hunter Medical Research Institute and The University of Newcastle, Newcastle, NSW, Australia

^2^University of Technology Sydney, Faculty of Science, School of Life Sciences, Sydney, NSW, Australia

^3^Telethon Kids Institute, Centre for Health Research, The University of Western Australia, Nedlands, Western Australia, Australia

^4^Centre for Inflammation, Centenary Institute and University of Technology Sydney, Faculty of Science, School of Life Sciences, Sydney, NSW, Australia

^5^Asthma and Airway Disease Research Center, University of Arizona

* = authors contributed equally

**Correspondence:**

Professor Phil Hansbro

Centenary Institute

Building 93 RPA, Missenden Rd

Camperdown, NSW 2050, Australia

Phone: +61 427 263 084

Email: Philip.Hansbro@uts.edu.au

Dr Chantal Donovan

University of Technology Sydney

15 Broadway

Ultimo, NSW 2050, Australia

Email: chantal.donovan@uts.edu.au

**Additional Methods**

**Bronchoalveolar lavage**

Bronchoalveolar lavage fluid was collected as previously described (S1-S4).

Mice were cannulated *via* the trachea and the left lobe of the lung flushed twice with 0.5 mL of PBS. Red blood cells were lysed, and remaining cells counted by trypan blue exclusion. Remaining cells were spun onto slides and stained with May-Grunwald Giemsa and differentially enumerated. A minimum of 200 cells were counted per sample.

**Mucus-secreting cells (MSCs)**

Lungs were perfused with 0.9% NaCl solution, inflated and fixed with 10% neutral-buffered formalin. Formalin-fixed lungs were then embedded in paraffin and sectioned longitudinally (3-5 μm per section). Four light micrographs of small airways taken at 40X magnification from Alcian-Blue Periodic Acid-Schiff (PAS) stained lung sections were randomly selected per mouse (S5-S7). The number of MSCs located in the airway epithelium was enumerated per μm of basement membrane length using Image J software (version 1.52a).

**Collagen deposition**

Collagen surrounding small airways was assessed using Sirius Red/Fast Green counterstained lung sections, whereby 6 light micrographs of small airways taken at 40X magnification were randomly selected per mouse, and total collagen area was manually outlined and calculated per μm of basement membrane length using Image J software (version 1.52a) (S4, S5, S7, S8).

**Lung function**

Airway hyperresponsiveness was assessed using the forced oscillation technique on a Scireq Flexivent FX1 machine as previously described (S9, S10). Briefly, mice were anaesthetized with 100 mg/kg of ketamine (Pamell) and 10 mg/kg of xylazine (Troy Laboratories) *via* intraperitoneal injection. Following cannulation of the trachea, mice were ventilated at a respiratory rate of 450 breaths/min. AHR was measured *via* instillation of increasing doses (0, 0.1, 0.3, 1, 3, 10, and 30 mg/mL) of nebulized methacholine chloride (acetyl-β-methylcholine chloride [MCh] in saline, #A2251, Sigma-Aldrich) and performing snapshot and prime-8 perturbations following the delivery of each dose.

**RNA extraction, bioanalysis, and RNA-sequencing**

Lung airway and parenchyma tissues were separated by blunt dissection under a dissection microscope as previously described (E3) and stored in RNA*Later*^TM^ stabilization solution (#AM7021, Invitrogen) overnight (4°C), then -20°C. For RNA extraction, samples were thawed, excess RNA*Later*^TM^ solution removed, transferred into 5 mL tubes containing 1 mL of ice-cold TRI Reagent (#T9424, Sigma-Aldrich), and RNA extracted as per the manufacturer’s instructions. RNA was then purified using RNeasy MinElute Cleanup Kits (Qiagen, #74204) according to the manufacturer’s instructions. After quantifying RNA yield and quality using a NanoDrop 1000 spectrophotometer (Thermo Fisher Scientific), samples with an optical density (OD) 260/280 ratio > 1.8 and an OD 260/230 ratio > 2 were assessed for RNA integrity using an Agilent 2100 Bioanalyzer System (#G2939BA, Agilent Technologies) according to the manufacturer’s instructions. Samples with an RNA Integrity Number (RIN) > 8 were sequenced.

RNA-sequencing (RNA-seq) was performed at the Australian Genome Research Facility (Melbourne, Australia) and data processed and analyzed as previously described (S11). Briefly, libraries were sequenced using a NovaSeq 6000 and 20 million 100 bp single reads were assessed for quality control using FastQC. RNA-seq raw sequence data were then processed using the ENCODE RNA-seq pipeline (<https://github.com/ENCODE-DCC/rna-seq-pipeline>). RNA-seq reads were aligned to the mouse genome (assembly: mm10, genome annotation: m21) using STAR (v2.5.1b), followed by gene quantitation with RSEM (v1.2.31) and Kallisto (v0.44.0).

**Differential gene expression and network analysis**

Differential gene expression and network analysis were performed as previously described (S11). Briefly, the R package Limma (v3.42.2) and voom function was used to calculate differential gene expression. Genes with low expression were filtered out from gene count data by only including genes with a count per million (CPM) equivalent of ≥ 10 in at least 9 samples. Normalization factors were calculated using the trimmed mean of M values (TMM) to eliminate composition bias between libraries, followed by voom transformation to convert the read counts to log2-CPM with associated weights, ready for linear modelling. P-values were adjusted for multiple comparisons using the Benjamini-Hochberg method, and those with *P* < 0.05 were considered significant. For differentially expressed genes in separate airway and parenchyma compartments, Gene Ontology (GO) analysis was performed using the R package clusterProfiler

The R package WGCNA was used to construct modules or clusters of genes with highly correlated patterns of gene expression within airway and parenchyma tissues separately. Raw gene counts were normalized using a variance stabilizing transformation and genes with < 10 counts per sample or without an official MGI symbol were excluded from analysis, resulting in 16,803 and 16,177 genes being used as input for the airway and parenchyma networks, respectively. Modules were functionally annotated using terms enriched in GO biological processes or pathways from the ReactomePA R package. For GO analysis, p-values were calculated using Fisher’s exact tests and Bonferroni-corrected, while for ReactomePA p-values were derived from hypergeometric tests with Benjamini-Hochberg correction, corrected p-values < 0.05 were considered significant.

To identify gene network modules associated with disease, the median adjusted p-value and median log2fold-change (Log2FC) of all genes within each module were used to rank differentially expressed modules for each tissue. Genes in each module with a module connectivity > 2 times the standard deviation from the mean and an adjusted p-value < 0.01 were deemed to be driver genes associated with that particular module. Transcription factors upstream of all driver genes within a module were predicted using ChIP-X Enrichment Analysis 3 (ChEA3) across 6 primary reference gene set libraries (S12).

**Additional References**

S1. Starkey MR, Plank MW, Casolari P, Papi A, Pavlidis S, Guo Y, Cameron GJM, Haw TJ, Tam A, Obiedat M, Donovan C, Hansbro NG, Nguyen DH, Nair PM, Kim RY, Horvat JC, Kaiko GE, Durum SK, Wark PA, Sin DD, Caramori G, Adcock IM, Foster PS, Hansbro PM. IL-22 and its receptors are increased in human and experimental COPD and contribute to pathogenesis. *Eur Respir J* 2019; 54.

S2. Liu G, Cooley MA, Jarnicki AG, Hsu AC, Nair PM, Haw TJ, Fricker M, Gellatly SL, Kim RY, Inman MD, Tjin G, Wark PA, Walker MM, Horvat JC, Oliver BG, Argraves WS, Knight DA, Burgess JK, Hansbro PM. Fibulin-1 regulates the pathogenesis of tissue remodeling in respiratory diseases. *JCI Insight* 2016; 1.S3. Haw TJ, Starkey MR, Nair PM, Pavlidis S, Liu G, Nguyen DH, Hsu AC, Hanish I, Kim RY, Collison AM, Inman MD, Wark PA, Foster PS, Knight DA, Mattes J, Yagita H, Adcock IM, Horvat JC, Hansbro PM. A pathogenic role for tumor necrosis factor-related apoptosis-inducing ligand in chronic obstructive pulmonary disease. *Mucosal Immunol* 2016; 9: 859-872.

S4. Donovan C, Starkey MR, Kim RY, Rana BMJ, Barlow JL, Jones B, Haw TJ, Mono Nair P, Budden K, Cameron GJM, Horvat JC, Wark PA, Foster PS, McKenzie ANJ, Hansbro PM. Roles for T/B lymphocytes and ILC2s in experimental chronic obstructive pulmonary disease. *J Leukoc Biol* 2019; 105: 143-150.

S5. Ali MK, Kim RY, Brown AC, Mayall JR, Karim R, Pinkerton JW, Liu G, Martin KL, Starkey MR, Pillar AL, Donovan C, Pathinayake PS, Carroll OR, Trinder D, Tay HL, Badi YE, Kermani NZ, Guo YK, Aryal R, Mumby S, Pavlidis S, Adcock IM, Weaver J, Xenaki D, Oliver BG, Holliday EG, Foster PS, Wark PA, Johnstone DM, Milward EA, Hansbro PM, Horvat JC. Crucial role for lung iron level and regulation in the pathogenesis and severity of asthma. *Eur Respir J* 2020; 55: 1901340.

S6. Thorburn AN, O'Sullivan BJ, Thomas R, Kumar RK, Foster PS, Gibson PG, Hansbro PM. Pneumococcal conjugate vaccine-induced regulatory T cells suppress the development of allergic airways disease. *Thorax* 2010; 65: 1053-1060.

S7. Nair PM, Starkey MR, Haw TJ, Liu G, Horvat JC, Morris JC, Verrills NM, Clark AR, Ammit AJ, Hansbro PM. Targeting PP2A and proteasome activity ameliorates features of allergic airway disease in mice. *Allergy* 2017; 72: 1891-1903.

S8. Liu G, Cooley MA, Nair PM, Donovan C, Hsu AC, Jarnicki AG, Haw TJ, Hansbro NG, Ge Q, Brown AC, Tay H, Foster PS, Wark PA, Horvat JC, Bourke JE, Grainge CL, Argraves WS, Oliver BG, Knight DA, Burgess JK, Hansbro PM. Airway remodelling and inflammation in asthma are dependent on the extracellular matrix protein fibulin-1c. *J Pathol* 2017; 243: 510-523.

S9. Kim RY, Pinkerton JW, Essilfie AT, Robertson AAB, Baines KJ, Brown AC, Mayall JR, Ali MK, Starkey MR, Hansbro NG, Hirota JA, Wood LG, Simpson JL, Knight DA, Wark PA, Gibson PG, O'Neill LAJ, Cooper MA, Horvat JC, Hansbro PM. Role for NLRP3 Inflammasome-mediated, IL-1beta-Dependent Responses in Severe, Steroid-Resistant Asthma. *Am J Respir Crit Care Med* 2017; 196: 283-297.

S10. Kim RY, Horvat JC, Pinkerton JW, Starkey MR, Essilfie AT, Mayall JR, Nair PM, Hansbro NG, Jones B, Haw TJ, Sunkara KP, Nguyen TH, Jarnicki AG, Keely S, Mattes J, Adcock IM, Foster PS, Hansbro PM. MicroRNA-21 drives severe, steroid-insensitive experimental asthma by amplifying phosphoinositide 3-kinase-mediated suppression of histone deacetylase 2. *J Allergy Clin Immunol* 2017; 139: 519-532.

S11. Tu X, Kim RY, Brown AC, de Jong E, Jones-Freeman B, Ali MK, Gomez HM, Budden KF, Starkey MR, Cameron GJM, Loering S, Nguyen DH, Mono Nair P, Haw TJ, Alemao CA, Faiz A, Tay HL, Wark PAB, Knight DA, Foster PS, Bosco A, Horvat JC, Hansbro PM, Donovan C. Airway and parenchymal transcriptomics in a novel model of asthma and COPD overlap. *J Allergy Clin Immunol* 2022.

S12. Keenan AB, Torre D, Lachmann A, Leong AK, Wojciechowicz ML, Utti V, Jagodnik KM, Kropiwnicki E, Wang Z, Ma'ayan A. ChEA3: transcription factor enrichment analysis by orthogonal omics integration. *Nucleic Acids Res* 2019; 47: W212-W224.

S13. Sikkema L, Strobl D, Zappia L, Madissoon E, Markov NS, Zaragosi L, Ansari M, Arguel M, Apperloo L, Bécavin C, et al. An integrated cell atlas of the human lung in health and disease. *bioRxiv* 2022:2022.2003.2010.483747.

S14. Karlsson M, Zhang C, Mear L, Zhong W, Digre A, Katona B, Sjostedt E, Butler L, Odeberg J, Dusart P, et al. A single-cell type transcriptomics map of human tissues. *Sci Adv* 2021, 7. Human Protein Atlas proteinatlas.org. <https://www.proteinatlas.org/ENSG00000066336-SPI1/single+cell+type/lung>; <https://www.proteinatlas.org/ENSG00000136689-IL1RN/single+cell+type/lung>; <https://www.proteinatlas.org/ENSG00000169403-PTAFR/single+cell+type/lung>; <https://www.proteinatlas.org/ENSG00000140678-ITGAX/single+cell+type/lung>; <https://www.proteinatlas.org/ENSG00000172243-CLEC7A/single+cell+type/lung>; v21.proteinatlas.org.

**Table S1. Airway and parenchyma network modules**

| **Airway modules** | **Associated function** |
| --- | --- |
| Red | Immune (innate) / inflammation |
| Green | Immune/T cell |
| Dark green | Regulation of metabolism / macromolecule modification |
| Turquoise | Immune/metabolism |
| Blue | Translation |
| Dark red | Complement/B-cell/Ig |
| Magenta | Cell cycle |
| Yellow | Developmental biology/angiogenesis |
| Purple | Regulation of development / vasculature development |
| Salmon | Developmental biology / adhesion |
| Green yellow | Mixed: RNA/DNA/protein functions |
| Cyan | Signaling/communication/structural development |
| Tan | Macromolecule/vesicle transport/localization/metabolism |
| Royal blue | Protein modification |
| Grey | Mitochondrial resp. electron transport chain |
| Light green | Mitochondrial translation |
| Brown | Cilium/organelle assembly |
| Black | Cilium activity |
| Pink | Muscle development / differentiation |
| Light yellow | Macromolecule localization |
| Light cyan | Acids/lipids metabolism |
| **Parenchyma modules** | **Associated function** |
| Light green | Cell cycle |
| Tan | Cilium/organelle assembly |
| Light cyan | Cilium/organelle assembly |
| Yellow | Immune/T cell |
| Magenta | Protein activity/transport/modification |
| Grey | Complement/B-cell/Ig |
| Blue | Immune (innate) / inflammation / metabolism |
| Brown | Regulation of biological process/signaling |
| Turquoise | Gene expression/translation |
| Green | Developmental biology |
| Red | Developmental biology/intracellular |
| Cyan | Molecule transport / localization |
| Light yellow | Adaptive immune |
| Dark red | Muscle contraction / development |
| Black | Transcription / RNA splicing |
| Green yellow | Acids/lipids metabolism |
| Salmon | RNA processing / gene expression |


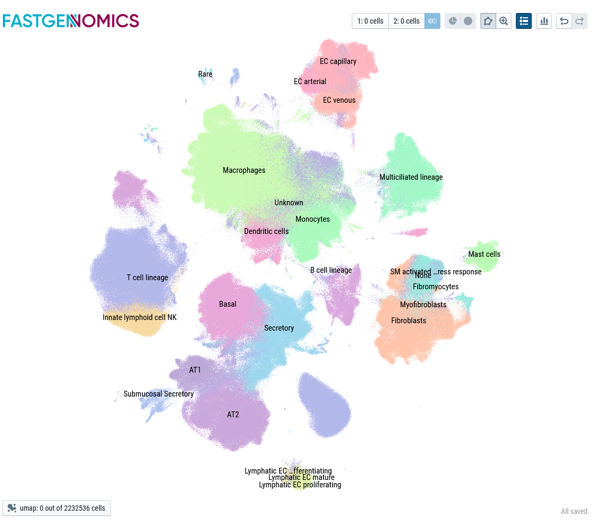

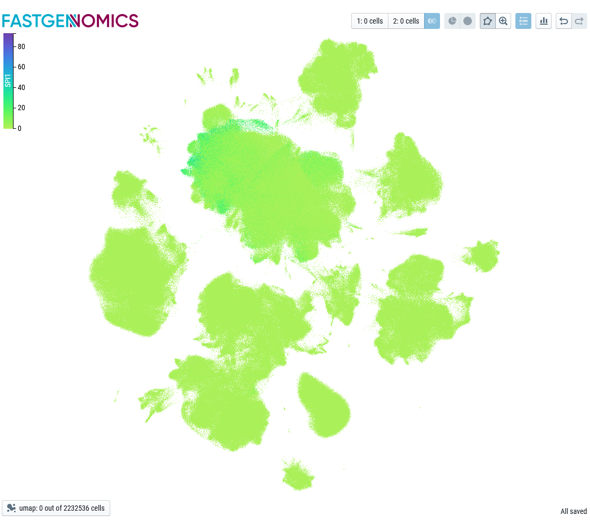


Figure S1: SPI1 overlayed onto the single-cell data from the integrated Human Lung Cell Atlas [E13].


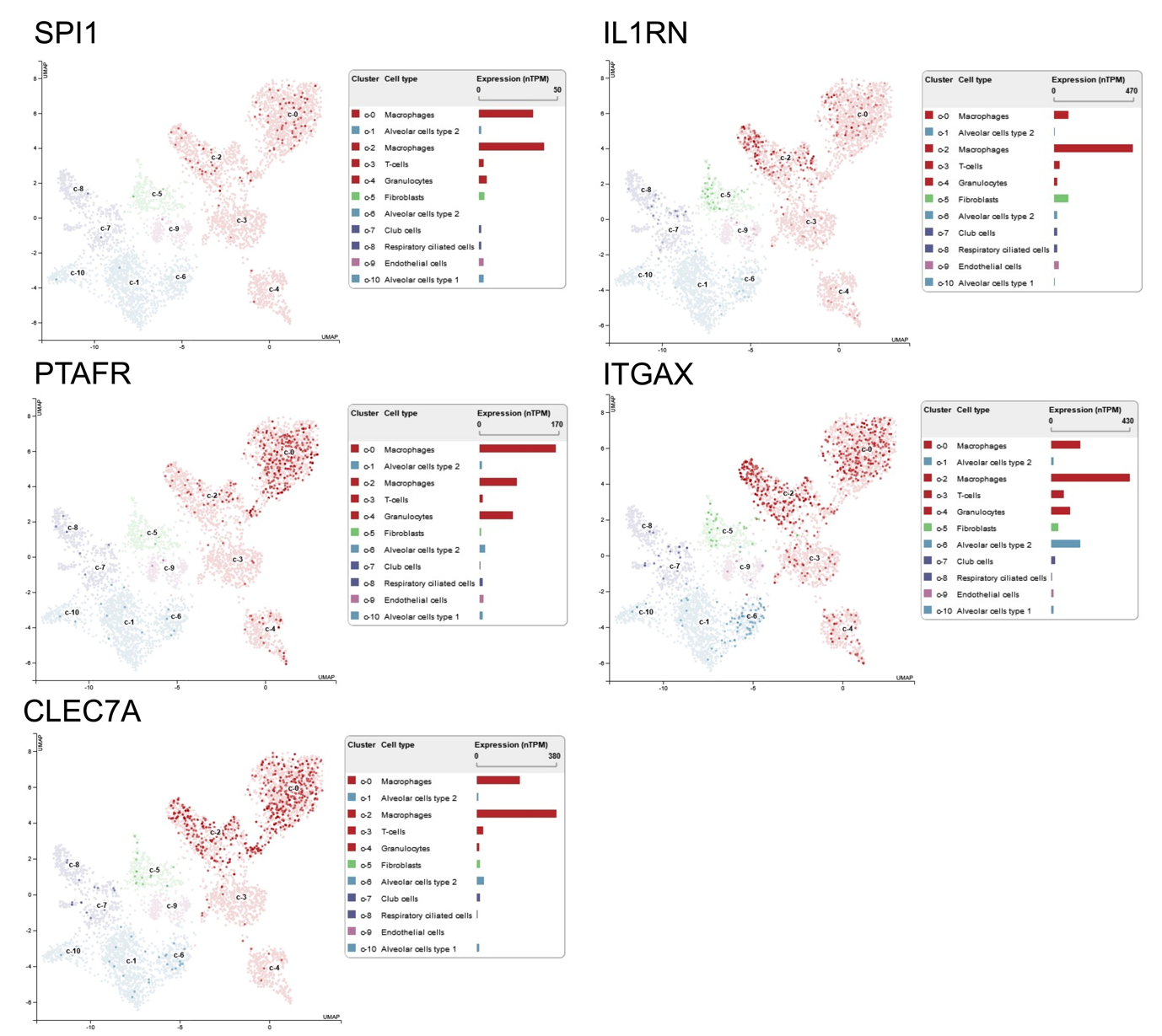


Figure S2: SPI1, IL1RN, PTAFR, ITGAX and CLEC7A overlayed onto the single-cell data from the integrated Human Protein Atlas [E14].
